# Doppler Measurement of Modulated Light for High Speed Vehicles

**DOI:** 10.3390/s22041444

**Published:** 2022-02-13

**Authors:** Kookjin Sung, Manoranjan Majji

**Affiliations:** Aerospace Engineering Department, Texas A&M University, College Station, TX 77843, USA; kookjin.sung@tamu.edu

**Keywords:** Doppler sensing, transimpedance amplifier, signal modulation, rate estimation

## Abstract

Technical details associated with a novel relative motion sensor system are elaborated in the paper. By utilizing the Doppler effect, the optical sensor system estimates the relative motion rates between the sensor and the moving object equipped with modulating light sources and relatively inexpensive electrical components. A transimpedance amplifier (TIA) sensing circuit is employed to measure the Doppler shift exhibited by the amplitude modulated light sources on the moving platform. Implementation details associated with the amplitude modulation and photo-detection processes are discussed using representative hardware elements. A heterodyne mixing process with a reference signal is shown to improve the signal-to-noise ratios of the Doppler shift estimation processing pipeline. Benchtop prototype experiments are used to demonstrate the utility of the proposed technology for relative motion estimation applications.

## 1. Introduction

Safe proximity operations of a moving vehicle are dependent on accurate relative navigation to drive the guidance and control loops. To this end, several relative position and attitude estimation systems have been developed for the navigation of vehicles [1]. The prevalent use of radio frequency (RF) modulation for communication, ranging and navigation paved the way for many advanced relative position and attitude estimation systems in engineering practice (cf. Chap. 3–6, 9 [1]). The global positioning system (GPS) technology enables the localization of a receiver that is able to receive localization signals from at least four space vehicles (SVs) [2,3]. With the advent of modern real-time kinematic tracking (RTK) capabilities, the GPS localization has reduced the typical localization error covariances from a few meters to within a meter. However, the precise RTK localization signal is only available at a low update rate (typical rates are of order of less than 1 Hz). Furthermore, the GPS sensing hardware only provides positional information of the sensor with respect to the geodetic reference frame. Integration of the position-fix from the GPS hardware with inertial sensing solutions (such as a 3 axis accelerometer and a rate-gyroscope that constitute most inertial navigation solutions (INS)) is necessary to provide a reliable position update capability between the high accuracy position fixes of the GPS sensor system. This represents the state of practice for navigation and avionics packages for autonomous systems that are currently in operation.

While the signals and navigation measurements obtained from the integration of the GPS and INS sensors form an acceptable positioning, heading and attitude solution for low frequency (less than 5 Hz) motion platforms, it is not acceptable for high-frequency relative motion platforms. High-frequency motions are typically experienced by platforms that exhibit faster motions such as unmanned aerial systems (UAS). UAS platforms routinely employ challenging and aggressive maneuvers to negotiate complex mission scenarios. This capability is also desired in self-driving automobiles when collision avoidance and passenger safety operations stipulate rapid reaction times. To this end, a variety of sensing solutions are used to augment the GPS-INS system in localization and pose estimation of autonomous systems. Furthermore, the use of ancillary relative positioning and navigation systems aids in catastrophic failures in the absence of GPS signals. A plethora of radio frequency (RF) solutions are used to augment the GPS-INS localization sensing systems. Proximity sensors installed on a modern car typify the type and kind of RF solutions employed by the engineers to augment the localization services provided by the GPS-INS technologies. Radar systems are routinely used to maintain speed control and formations in automotive tracking systems. The modern automotive radar system is a chipset type sensor consisting of multiple transmit and receive channels that provide the angular information about the target by determining the phase difference between the signals. The automotive radar sensors are classified into short-range radar (SRR) (≈30 m) and long-range radar (LRR) (≈150 m) according to their capability of range detection. These sensors can provide the instantaneous radial velocity of a moving object. To accommodate wider bandwidth demands, a 77 GHz sensor is being used [4]. In the field of self-driving automobile research, SRR sensors are designed for parking/lane-change assistance and blindspot detection because it offers a wide field of view. LRR sensors operate at ranges of more than 100 m, suitable for adaptive cruise control. LRR sensors mostly adopt the 77 GHz band in order to take advantage of better resolution [5,6,7].

Vision-based navigation systems form an important alternative to radio spectrum for relative navigation sensor systems. VisNav is an electro-optical sensing system that uses a set of active beacons mounted at known locations on the structure to image a position-sensitive photodetector (PSD) [8,9]. The PSD acts as an imager and produces a voltage output proportional to the location of the incident energy on both axes of the PSD. Since it is an analog system, the PSD operates at a kHz range and also facilitates the beacon identification, as each beacon module has a unique frequency [8,9]. A digital version of this sensor was developed in subsequent work by Wong and Majji [10]. In the digital structured light system, the PSD is replaced by a digital camera, and the frame-rate of the camera is utilized for simultaneous beacon identification and relative pose estimation processes. An automated approach for beacon synchronization with the camera is also proposed, which is used periodically to reset the beacons and implement the power amplification circuit of the beacons as a function of the estimated range from the camera. This power amplification system enables the camera to have the right amount of exposure by varying the power emitted by the beacons.

While the direct sensing of the relative pose between the structured light beacons is of great utility in guidance and control of vehicles executing six degrees of freedom (DOF) maneuvers, an estimate of the relative angular velocities and translational speeds is useful for more aggressive maneuvers in autonomous systems. While the rate information can be inferred if the relative pose can be estimated at sufficiently high update rates using a Kalman filter that makes use of a simple kinematic model for relative motion dynamics, the direct sensing of rates enables estimation of the rates with a much higher bandwidth and better signal-to-noise (SNR) characteristics. Inertial measurement units (IMUs) on the vehicles involved in the proximity operations provide sufficient signals for relative pose-rate estimation in the cooperative proximity operations. However, in non-cooperative applications, the inertial sensors do not have the ability to measure the relative translational rates between the bodies involved. This is because the rate gyroscopes only measure the angular velocity vector of the bodies involved with respect to an inertial frame. Complementary rate sensing technologies are useful to augment the effect of systematic drifts incurred by the inertial sensor systems. Direct estimation of the rate also enables accurate relative pose estimation by integration from the best estimated relative pose. The multi-sensor integration involved by combining different sensing modalities is an important element in the automation of vehicular proximity operations.

Modulated electromagnetic signals modulated periodically experience a Doppler shift when the waveform emitter and receiver experience relative velocity with respect to each other [11]. The amount of shift is related to the speed of the motion of the emitter, and the sign of the shift dictates the relative direction of motion of the emitter and receiver pair. Ultrasonic and radio frequency signals are commercially used to sense the rates of motion between the transmitter/receiver pairs. Additionally, light waves can also be used as the carrier wave for Doppler shift detection and rate estimation. This paper uses light as a signal carrier. Optical modulation has some distinct advantages for vehicle navigation applications. (i) Exclusive bandwidth from existing bandwidth of RF signals, avoiding interference with them; (ii) less power and mass are required for implementation; (iii) it is seldom intercepted or detected in the middle of propagation; (iv) effective in ambient temperature [12]. To this end, several sensor systems for relative velocity have been developed [13,14,15,16,17]. Previous works focused on measuring frequency shifts of light using a laser beam with highly precise reflection structures. For example, laser Doppler velocimetry (LDV) is commercially used in the field of fluid dynamic investigations and medical imaging for blood flow. The proposed proximity sensor is distinguished from previous sensors in certain aspects. The sensor chooses an LED as an optical source to take advantage of its high linearity that allows a light level to easily be controlled by input voltages. The growth of LED technology has enhanced its optical emission power and sensibility, which highly affect stable modulation. For detection applications, LED can be a competitive signal source in that its emission frequency can be controlled by modulation. Implementing an LED modulation is more economical than laser modulation, and its exposure to human eyes is less hazardous than a laser beam. Moreover, the proposed sensor uses the one-way travel of the light beam and it does not require reflection. The sensor is installed on an observer, while the signal source is placed on a moving object. The LED light typically propagates in free space with a wider angle range than the laser does, and complex scattering patterns are present when reflected. Possible interference between scattered beams is avoided by the practice of a one-way path. This paper focuses on relative rate sensor technologies that use the Doppler shift caused by relative motion of optical elements. To this end, light-emitting diodes (LED) are used for carrier signal modulation, and an avalanche photodiode (APD) with matching bandwidth is employed to build prototype sensor components. While the use of commercial LED light sources offers some constraints on the operating distance, dynamic range and resolution of the prototype instrument, it demonstrates the feasibility of the sensing technology and a systematic template to the underlying research issues for scalability to practical sensor implementations. We hasten to point out that the proposed sensor solution can be adapted to use laser diodes as opposed to LEDs to extend the operating range of the instrument. The paper is laid out as follows. After a review of the principles of Doppler shift detection in radio ranging applications, a brief discussion on the technologies involved in photonic Doppler sensing is presented, along with the specific challenges on the sensitivities of the relevant components and their characteristic features and implementation considerations. The relative pose rate sensor concept is then discussed, along with a technical discussion on the benchtop prototype and related testing apparatus developed at our laboratory to characterize the sensor prototype. Discussions pertaining to the scalability of the benchtop prototype, its limitations, operational constraints and considerations are then elaborated. Experimental results obtained by using the breadboard prototype are then discussed. The paper concludes with a brief discussion on the scalability of the proposed technology into sensor hardware of higher dynamic range, sensitivity, resolution and precision.

## 2. Optical Waveform for Doppler Radar Application

### 2.1. Principles of Doppler Radar

The Doppler effect is observed when a relative motion takes place between the vehicle and the observer. A Doppler radar acquires the velocity of the moving target along the line of sight vector by observing how the target shifts the radio frequency (RF) signal transmitted by the radar. Since the actual speed of a vehicle is incomparably smaller than the speed of light, the radar is required to modulate the transmitted RF signal. In the presence of a target within the radar’s operating range, the transmitting signal with modulating frequency $f_{o}$ hits the moving target and a delayed replica of the transmitted waveform is received by the radar antenna.

The transmitted signal is a coherent sinusoidal wave with frequency of $f_{o}$

$$S_{t}\left(t\right)=A_{1}\;\cos\left(2\pi f_{o}\;t\right)$$

The received signal is delayed by τ=r/c in which *r* is the radial distance to a target and *c* is the light speed.

$$\begin{array}{cc} S_{r}\left(t\right) & =A_{2}\;S_{t}\left(t-\tau \right)\\ \; & =A_{1}\;A_{2}\cos\left[2\pi f_{o}\;\left(t-\tau \right)\right]\\ \; & =A_{1}\;A_{2}\cos\left[2\pi f_{o}\;\left(t-\frac{r}{c}\right)\right] \end{array}$$

The perceived frequency, $f_{s}$, is the rate of change of the phase of the received signal [18],

(1) $$f_{s}=\frac{d}{dt}\;\left[f_{o}\;\left(t-\frac{r}{c}\right)\right]=f_{o}-\frac{f_{o}}{c}\;\frac{dr}{dt}$$

Then, the frequency shift can be expressed

$$\Delta \;f_{o}=f_{s}-f_{o}=-\frac{f_{o}}{c}\;\frac{dr}{dt}$$

**Figure 1:**
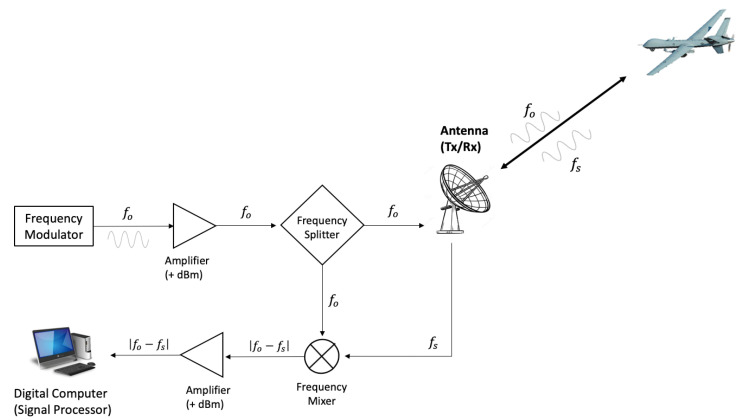
Doppler radar system.

In this paper, the sensor holds that a radar is stationary on the ground and the target of interest has a constant velocity coming forward to the radar.

(2) $$\Delta \;f=f_{s}-f_{o}=V_{rel}\;\cos\theta \;\frac{f_{o}}{c}$$

where $V_{rel}\cos\theta =-dr/dt$ is the relative speed of a vehicle to the sensor and θ is the elevation angle of the antenna, also known as the boresight angle [19,20]. A frequency mixer compares the received signal and the reference original signal so that the interference pattern of two signals creates a beat signal whose frequency is the value of the shift. This process is known as heterodyne interferometry. The operation of the Doppler radar is illustrated in Figure 1.

Typical RF radars modulate the transmitting signals at a very high frequency (VHF) band (30–300 MHz) to super-high frequency (SHF) band (3–30 GHz). For example, to detect very slow motion, traffic speed guns use an extremely high frequency (EHF) band (30–300 GHz) that even record the speeds of pedestrians. While the principles of the Doppler radar are pretty commonly used in RF applications, other electromagnetic waves can also be used in interferometric applications. In this paper, light modulation is considered as the primary modality of interferometric energy transmitters for Doppler sensing.

### 2.2. Photonic Doppler Sensing

All incident light must be converted to electrical signals, transmitted, received and processed. Our navigation system utilizes light to deliver the Doppler range rate information of the aircraft to the sensor. With the use of light, the sensor can take advantage of prompt data transmission and function safely without the risk of electromagnetic interference on electronics.

#### 2.2.1. Optical Modulation

**Figure 2:**
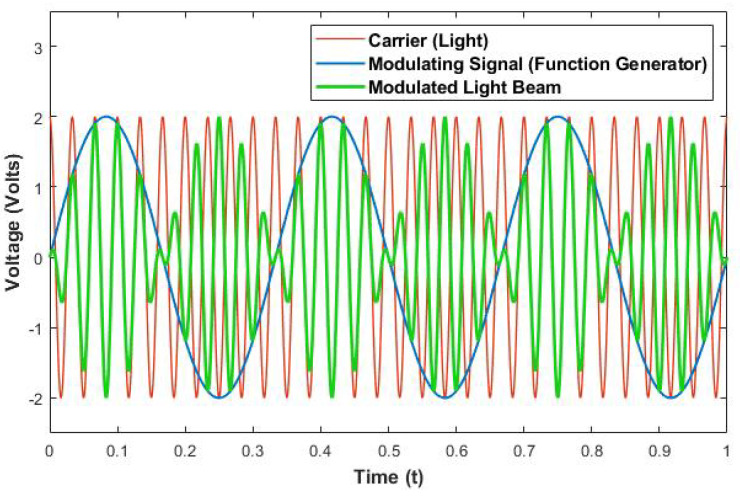
Amplitude modulation of signal: When the light source is excited by an external electrical signal (blue), an amplitude of carrier wave (red) is modulated in proportion to the electrical signal and generated in the form of the modulated signal (green).

Light is an electromagnetic wave with a frequency of 430 THz far beyond the frequency resolution of photodetectors [21]. Therefore, the signal must be down-converted into a much smaller baseband using amplitude modulation so that the detection and signal processing system can handle the phase shift experienced by relative range rates in operating vehicles. In amplitude modulation, the voltage level of the external electrical signal modulates the amplitude of the carrier signal, which is light for optical modulation. The envelope of the modulated light beam along with the contours of the modulating signal are shown in Figure 2.

$$\begin{array}{cc} M(t) & =\;M\cos(2\pi f\;t)—\mathrm{Modulating}\;\mathrm{Signal}\\ C(t) & =\;C\cos(2\pi f_{c}\;t)—\mathrm{Carrier}\;\mathrm{Signal}\\ A(t) & =\;[A+M(t)]\;C(t)—\mathrm{Amplitude}\;\mathrm{Modulated}\;\mathrm{Signal} \end{array}$$

For data transmission, both optical sources and detectors should operate within the detector’s frequency resolution. There are two main types of optical signal sources; light-emitting diodes (LED) and light amplification by stimulated emission of radiation (laser) diodes. LEDs are the economical and lower-performance sources used in applications where low data rates and/or short distances are anticipated. Laser light sources operate at much higher speeds, dissipate greater power levels, and require temperature compensation or control to maintain specified performance levels. Laser light sources also support long distance propagation. Photodetectors are also categorized into two main categories—PIN photodiodes and avalanche photodiodes (APD). PIN photodiodes are more commonly used, especially in less constricted applications. On the other hand, APD has properties of very high sensitivity and gain although it requires a much higher operating voltage than a PIN photodiode. With a high specification in gain, APD can be utilized for low light level measurement at high speed. For prototyping the proposed Doppler sensor, LED and APD are chosen as the light transducer and photodetector, respectively. This is because the LED beam loses optical power in a way that is inversely proportional to the square of the range, and APD is more capable of detecting the attenuated light energy.

An advantage of LED modulation is that its direct modulation shows high linearity between an input voltage to LED and the modulated output [22]. An analog modulation of LED shows high linearity with respect to the input electrical signal. An LED does not require a threshold current, while a laser typically remains inactive until it reaches a certain gain level. The direct input of time-varying electric current to LED can produce an optical signal without phase delay or clipping. Thus, the power driver of LED can be built in a simpler manner than the laser. This is useful at the prototype stage. The modulation bandwidth of the LED can be defined by the frequency where the power transfer function falls by 3 dB [23]. The frequency response of the LED relies on the injected carrier lifetime in the recombination region, $\tau_{i}$, and parasitic capacitance of the LED [24]. For LED modulation, the bandwidth based on the electrical aspect is used. This is because the output light power varies linearly with the input electric current [25]. Typically, high performance LEDs have a bandwidth of 60 to 80 MHz. This means that an emitted waveform experiences unacceptable delays at the higher frequency bands. This problem is caused when the input modulation outweighs the LED’s blinking resolution related to the rise and fall times of emission. When the input signal to LED reaches a high frequency, the emission response in the LED is unable to follow the changes, thus the emitted power becomes smaller. Rise time is the time required to rise from 10% to 90% of the peak of the light emission and the fall time is required from 90% to 10%. Noise is a natural phenomenon and inevitable part of high-frequency signals in electronics. Every electronic component experiences a random vibration of electrons and subtle temperature change that cause noise. There are several factors that create noise in the optical Doppler sensor.

- Thermal noise (Johnson noise): This unavoidable noise is generated by random thermal motion of charge carriers. It is generated regardless of the voltage applied.
- Flicker noise: The flicker electronic noise has a PSD (power spectral density) of 1/(frequency). This is a low-frequency phenomenon because higher frequencies are overshadowed by white noise. Typically, this noise affects the DC components of the circuit.

#### 2.2.2. Doppler Shift in Modulated Light

Measuring the optical Doppler shift can be quite complicated. Unlike sound and radio waves, the light sources do not usually propagate in a uniform manner to constrain the noise level far below the true signal. The frequency shift of modulated light can be explained by the group Doppler effect [26,27].

When an optical signal propagates, the frequency of the $n^{th}$ harmonic of the field, $\omega_{\pm n}$, can be described as

$$\omega_{\pm n}=\left(\omega_{o}\pm n\;f_{m}\right)\left(1+\frac{V}{c}\right),\;\;n=1,2,3\cdots$$

where $\omega_{o}$ is the central carrier frequency and $f_{m}$ is the modulation frequency. Thus, the difference between the shifted center frequency of carrier (*n* = 1) and the shifted side of frequencies is

$$\Delta \omega =f_{m}\left(1+\frac{V}{c}\right)$$

It means that the modulation frequency $f_{m}$ is also shifted to $f_{sm}$ by the Doppler effect proportional to the relative velocity, maintaining the amplitude of spectrum [28].

$$f_{sm}=f_{m}\pm \frac{V}{c}$$

The modulated frequency within the frequency resolution may be far less than that of typical RF-based Doppler radars. Therefore, the speed of a target affects the signal-to-noise (SNR) ratio of the Doppler circuit. If a target moves with pedestrian speed, the Doppler sensing in the MHz range will not result in a measurable frequency shift. However, if the design range of the sensor system is to detect speeds between 5 and 200 m/s, the shift outcomes are able to avoid the flicker noise that hinders low-frequency measurements.

#### 2.2.3. Modulation Bandwidth of LED

The modulation bandwidth of the illumination source is determined as the point where the signal power P(ω) falls to half its DC bias (zero frequency) power, $P_{0}$ as frequency increases. The modulation bandwidth of LED largely depends on the recombination lifetime, $\tau_{i}$, of the device [25]. If *R* is the electrical resistance and i(ω) is the current at the frequency ω, the power P(ω) is calculated by $P\left(\omega \right)=i^{2}\left(\omega \right)\;R$.

The ratio of the output power at the frequency ω to the power at zero modulation is written as

(3) $$Ratio\;\left(\mathrm{dB}\right)=10\log_{10}\frac{P(\omega )}{P(0)}=10\log_{10}\frac{i^{2}\left(\omega \right)}{i^{2}\left(0\right)}$$

The electrical 3-dB bandwidth takes place at the frequency point where P(ω)/P(0)=0.5 [25]. The 3-dB bandwidth indicates the maximum possible range of modulation in which the signals do not mutually interfere.

#### 2.2.4. Trans-Impedance Amplifier (TIA): Operation and Noise

**Figure 3:**
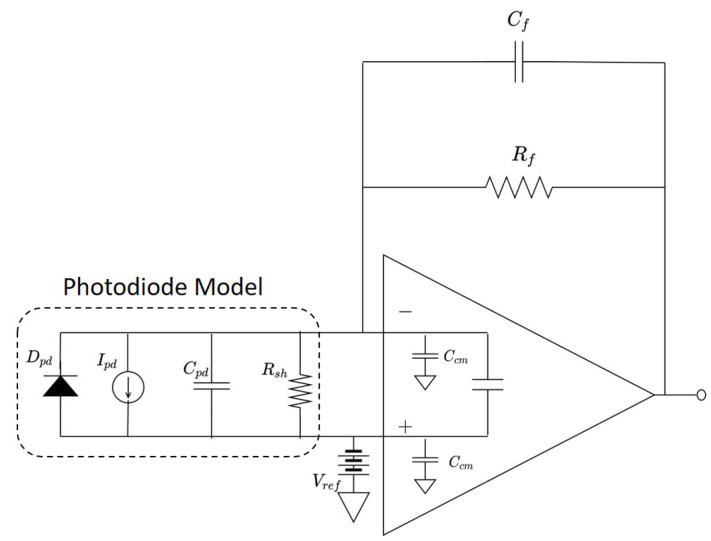
Transimpedance amplifier.

In order to take advantage of high-linearity, low DC level (zero frequency component), high gain and wide bandwidth, the proposed sensor employs a trans-impedance amplifier (TIA) integrating an avalanche photodiode. As illustrated in Figure 3, TIA consists of an operational amplifier (OpAmp) and passive electrical components, and the output gain is decided by the value of the feedback resistor. TIA conducts a current-to-voltage conversion and amplifies the voltage level according to the given gain. Therefore, in constructing a TIA system, the robustness of the desired sensor output against the noise should be carefully studied. The circuit’s noise behavior results from the kind of OpAmps used, passive electronic components, input noise current and noise voltage.

The frequency response of the TIA shows where the signal power gain is maximized and noise gain is minimized. A greater value of $C_{f}$ would be used to limit the bandwidth and reduce the voltage noise at higher frequencies [29]. This frequency band can be adjusted by feedback components ($R_{f}$ and $C_{f}$) and an input capacitance ($C_{i}=C_{pd}+C_{cm}$) described in Figure 3. The frequency response of the TIA is characterized by open-loop gain ($A_{ol}\left(s\right)$), input impedance ($Z_{i}\left(s\right)$), feedback factor (β(s)), pole frequency ($f_{p}$) and zero frequency ($f_{z}$) [21].

(4) $$\begin{array}{cc} Z_{i}\left(s\right) & =\frac{R_{f}}{1+sR_{f}\left(C_{f}+C_{i}\right)}\\ \beta (s) & =\frac{1+sR_{f}\;C_{f}}{1+sR_{f}\left(C_{f}+C_{i}\right)}\\ f_{p} & =\frac{1}{2\pi \;R_{f}C_{f}}\\ f_{z} & =\frac{1}{2\pi \;R_{f}\left(C_{f}+C_{i}\right)} \end{array}$$

Flicker noise $f_{f}$ is present until a certain level, which is typically around 100 Hz. The 1/β response represents noise gain, and the TIA gain can be described as

(5) $$G\left(s\right)=\frac{V_{o}}{I_{in}}=-Z_{i}\frac{A_{ol}\left(s\right)}{1+\beta \left(s\right)\;A_{ol}\left(s\right)}$$

**Figure 4:**
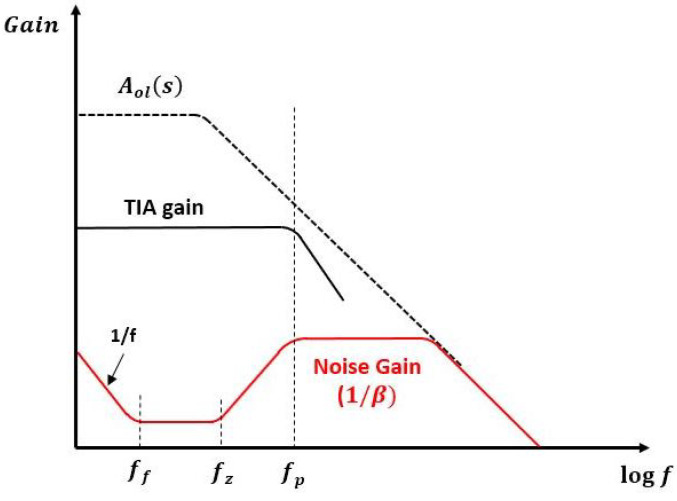
The frequency response of TIA and noise.

The noise curve begins to increase from $f_{z}$ with a slope of 1/β developed by feedback components and an input capacitance of an amplifier. The zero created by $C_{f}$ counterbalances an effect of a pole produced by $C_{i}$ at $f_{p}$ where this effect levels off a slope of 1/β [21]. According to the frequency response (Figure 4), having a modulation frequency between $f_{f}$ and $f_{z}$ is the best way to maximize the SNR of TIA. For the optical Doppler sensor, noisy signals caused by the LED, photodiode and TIA circuit are not negligible.

## 3. Sensor Design

### 3.1. Sensor Description

**Figure 5:**
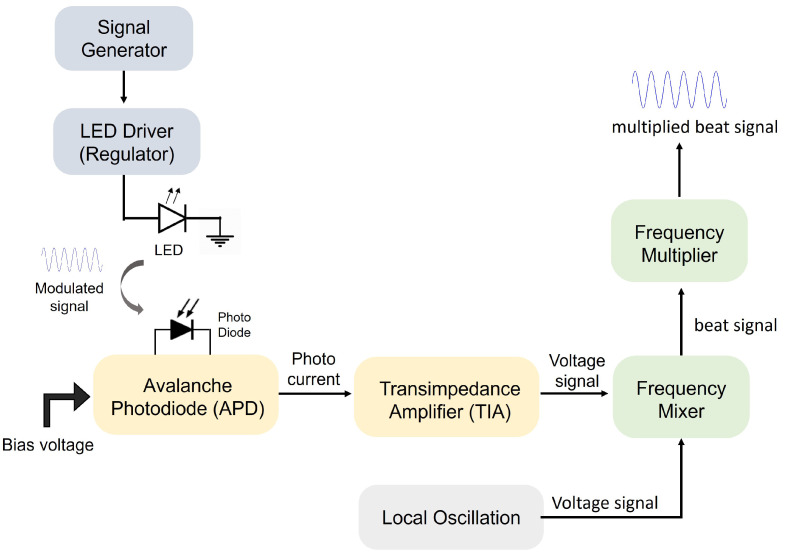
Diagram of the system setup for frequency shift measurement.

The proposed Doppler sensor consists of the light source (beacon) affixed to a moving vehicle (target) and photodetection sensor on a station along platform. The beacon emits amplitude modulated light with distinct modulation frequency $f_{i}$ toward the sensor. That is, the beacon generates sinusoidal modulating waveform $A_{i}\cos\left(2\pi f_{i}\right)$. When the target approaches the sensor, the movement with the modulation frequency $f_{i}$ is shifted in proportional to the relative velocity between the sensor and the target. The shifted frequency, $f_{i}^{\prime }$, is detected by TIA, which converts the incident photons to electrical signal, so that a signal processor is capable of analyzing them. The original transmitting frequency $f_{i}$ is a known value, and the sensor includes a function generator that creates a sinusoidal of $f_{i}$. This reference signal is transferred to the local oscillator (LO) port of the frequency mixer and the mixer’s RF port is connected to the output of the TIA sensor, so that the frequency-shifted waveform is transferred to it. The output port of the mixer yields the sinusoidal signal with the frequency of $\left|f_{i}^{\prime }-f_{i}\right|$ and thus the digital computing processor makes use of the beat components. In the digital computer, analog electrical signals should be digitized by analog-digital-conversion (ADC) implementation. Then, the discrete Fourier transform (DFT) on these quantized data enables us to read the numeric values of frequency shift. Using the mathematical relation between the Doppler shift and the relative velocity of a target, the relative velocity along the line sight vector is determined. When the modulated light is sensed by the sensor, this signal activates a photodetector, TIA, frequency mixer and frequency multiplier to reach the processor, as shown in Figure 5.

#### 3.1.1. Photodetector

There are two different photodiode configurations: photoconductive and photovoltaic mode. The photovoltaic detector is a zero bias voltage mode in which current is generated as a result of the absorption of photons of a voltage difference across a p–n junction. The photoconductive mode uses an external reverse bias voltage to a photodiode to increase electrical conductivity and expedite response time by increasing the width of the depletion layer when photons are absorbed [21]. For the optical Doppler sensor, an avalanche photodiode (APD) in the photoconductive mode is used as the light detector and the current source. Typically, APD requires a reserve bias voltage of 100–200 V. APDs are widely used in fiber optic systems to convert absorption of incoming photons into electrical form. The advantages of the APD over the PIN photodiode are the higher sensitivity and faster responsiveness. APDs achieve a significant level of signal-to-noise ratio (SNR) with an avalanche multiplication function inside the photodiode. Hence, APDs are suitable for applications of low-level light detection and high accuracy range finders. In this prototype, Hamamatsu’s Si APD S2384 is utilized for a photodetector provided with a reverse voltage of 180 V by the voltage amplifier.

#### 3.1.2. Transimpedance Amplifier (TIA)

**Figure 6:**
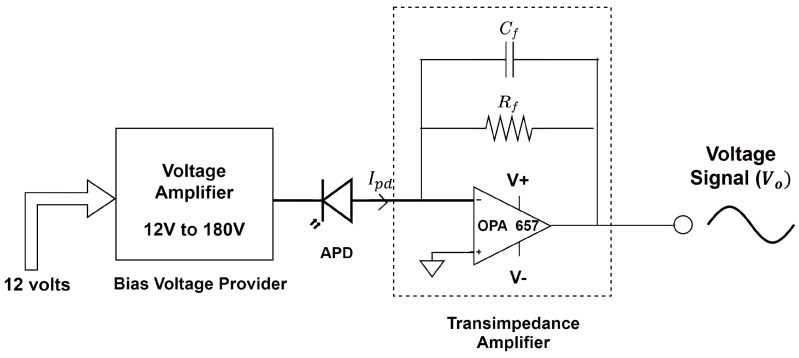
TIA consists of APD (S2384) and OpAmp (OPA 657) to convert the induced photo current to the voltage signal. The APD bias voltage is fed by voltage amplifier integrated to the TIA circuit. A 1 MΩ resistor and 0.1 pF capacitor are used for feedback resistance $R_{f}$ and feedback capacitance $C_{f}$, respectively.

The output current of the photodiode is transformed to voltage for use by subsequent sensor electronics by the TIA. Implementation details of the TIA used for light-based Doppler range rate sensing are now described. Note that the objective of this description is to provide a representative description of all the electronic considerations involved in the development of the prototype. For the Doppler sensor, TIA action is modeled as a linear relation of an output voltage ($V_{o}$) equal to the diode current ($I_{pd}$) times the feedback resistance ($R_{f}$), $V_{o}=I_{pd}\;R_{f}$. This gain defined by $V_{o}/I_{pd}$ is controlled by adjusting the feedback resistor $R_{f}$, and the bandwidth is controlled by $R_{f}$ and the feedback capacitor $C_{f}$. Moreover, a TIA isolates the load voltage swing from the photodiode capacitance for improved bandwidth. To avoid an undesirable 45-degree phase margin, the TIA, which is the second-order system, is required to adjust its poles and zeros with an appropriate choice of $R_{f}$ and $C_{f}$ [30]. The use of a photoconductive mode significantly improves the response speed and linearity of the device. Although reverse bias voltage can increase the dark current, a low pass filter connected to the output node of TIA diminishes the noise raised by a dark current. The schematics of TIA used for the proposed sensor are shown in Figure 6.

The employed TIA includes the high voltage module and a single gate junction field-effect transistor (JFET) ‘OPA657’ as an OpAmp. The voltage module provides a 150 V bias voltage to the APD and the OpAmp is fed by a 1 MΩ feedback resistor to achieve the gain of $10^{6}$. OPA657 offers a very high gain–bandwidth product, which allows greater than 10 MHz signal bandwidths up to gains of 44 dB with low input bias current. To ensure stability, the 0.1 pF capacitor is chosen for the feedback capacitance. Owing to the outstanding performance of an APD, the TIA used by the Doppler sensor obtains higher voltage outputs with a smaller circuit volume. The OpAmp OPA657 and its feedback components are integrated into the voltage amplifier module that amplifies the external 12 volts power supply up to 180 volts to feed the bias voltage of the APD. Hence, the APD is operational when it is connected to the TIA and the excited photo current signal $I_{pd}$ is converted to the voltage signal $V_{o}$ at the output port.

The frequency of the waveform observed at the output of the TIA is shifted from $f_{o}$, which is the modulating frequency at the light source. We utilized the frequency mixer to extract the shift by mixing the output of the TIA and the local oscillation whose frequency is $f_{o}$. For the mixer, SBL-3+ designed by Mini-Circuits is chosen. SBL-3+ is a plug-in type frequency mixer that performs from DC level (zero frequency) to 200 MHz with an excellent conversion loss, typically 4.81 dB. The modulated signal source to LED is generated by an arbitrary function generator, AFG1062 by Tektronix. Since AFG1062 does not provide a frequency level of the VHF band, the other function generator is used from the VHF transmitted signal. The voltage controlled oscillator ZOS-200+ with the linear amplifier ZX60-33LNR-S+, both designed by Mini-Circuits, are utilized. For a consistent input environment, the voltage setup of AFG1062 is equalized to the output of the linear amplifier. The measured frequency shift can be too small to obtain a coherent waveform within the sweep time. A frequency multiplier allows us to obtain several output signals. For the proposed sensor, TB-RMK-7-81+ by Mini-Circuits is embedded to the output of the function generator. TB-RMK-7-81+ is an evaluation board of RMK-7-81+, which uses a surface mount technology (SMT) type *X*7 multiplier used to transfer output frequencies to a band seven times higher. The multiplier board includes a Schottky diode and compatible filter circuit to achieve a low conversion loss as well as high rejection of irrelevant harmonics near its X7 output. To remove the unwarranted harmonics at high bandwidth, the active low pass filter is connected at the output of the TIA sensor. The low pass filter is designed to have a 3-dB attenuation at 500 MHz with very low signal loss.

### 3.2. Signal Processing

**Figure 7:**
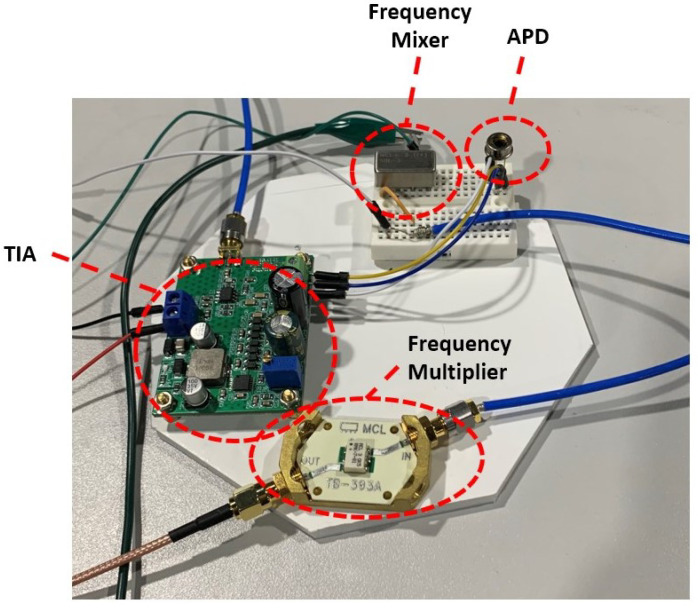
An image of the TIA sensor prototype and its components. On the TIA board, the feedback resistance is adjusted by the potentiometer, and the capacitors are soldered.

**Figure 8:**
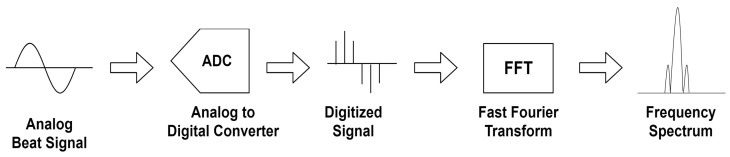
Signal processing schematic.

The output of the circuit in Figure 7 is the multiplied beat signal, and it is fed to the digital processor. Since the frequency of the beat signal indicates the frequency shift of the emitted optical signal induced by the relative motion, we need to apply Fourier transform to analyze its frequency component. An analog-to-digital converter (ADC) should be implemented first to convert the analog beat signal to digital format. Once the signal is sampled by ADC, the processor operates a Fourier transform that conducts time-to-frequency domain transforms to identify the frequency of decomposed sinusoidal components. The dominant frequency is chosen from the resultant spectrum and sent to the velocity estimation process. Figure 8 illustrates how the signals are processed through the Doppler sensor from the emission of the beacons.

In this project, we utilized the built-in ADC function in the digital oscilloscope. The employed digital oscilloscope is equipped with a 12-bit resolution ADC that returns the digitized data of sinusoidal waveform. The oscilloscope is connected to the output of the multiplier and records the digitized data while conducting the experiment. We used these data to extract the dominant frequencies and estimate the velocity of the relative motion as a post-processing. In the digital computer, fast Fourier transform (FFT) is carried out to calculate the dominant frequency of the stored data. Our FFT implementation is based on the discrete Fourier transform (DFT) of the frequency domain points, X(k), that are computed from the time domain points, x(n), in the record. The DFT calculation is written as

(6) $$X\left(k\right)=\frac{1}{N}\sum_{n=0}^{N-1}x\left(n\right)e^{-j\pi kn/N}$$

where *N* is the number of input samples, and *n* and *k* are the index of time samples and index of frequency point, respectively. The calculation returns a two-sided spectrum in complex form and each output point has the real (*R*) and imaginary (*I*) part. The processor computes the power spectrum (*P*) of each output point in dBm by the following equation.

(7) $$P=10\log_{10}\frac{P^{2}+I^{2}}{N}$$

The dominant frequency is obtained by the index value of the greatest peak of the power spectrum in the FFT domain and its corresponding frequency value. The beat signal is generated as a sinusoidal format by the frequency difference between the local oscillation and the received signal. To extract the frequency of the beat signal, the processor utilizes FFT implementation and determines the dominant frequency of the beat signal. Since the output of the mixer directly denotes the Doppler shift proportional to the relative velocity of a target, the velocity is obtained by simple conversion. It is noted that a separately embedded system processing pipeline needs to be incorporated in a sensor to replace this process for real-time signal processing. Optical signals from the beacon collected by the sensor are subject to ADC and FFT processing to obtain the numerical value of the Doppler shift. Although the output of the mixer does not specify the sign of the Doppler shift, it can be accessed contextually. In embedded system applications, these signal processing operations are carried out by dedicated computing elements.

## 4. Experiment

### 4.1. Benchtop Experiment Setup

**Figure 9:**
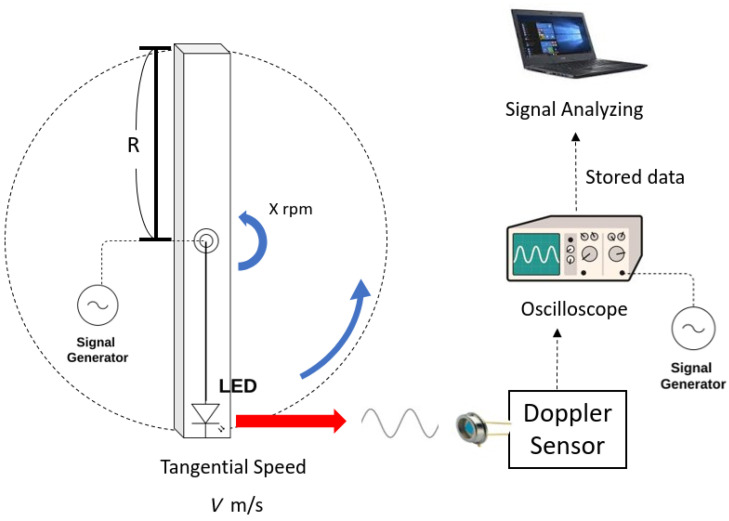
Schematic of the experiment setup for experimental testing of the Doppler rate sensor: The rotating wheel provides high tangential speed relative to the sensor.

To evaluate the functionality of TIA’s sensing ability, the measurement along the one-way travel of a signal is performed. A single LED operation is used in the experiment. It is assumed that the characterized single LED performance can be replicated to a multi-beacon operation. The proposed sensor requires substantial linear velocities to derive the generations of Doppler shift detectable in the laboratory. Therefore, a tangential velocity of circular motion is used to generate the required Doppler shift. A fan blade driven by a high revolution-per-minute (RPM) DC motor is set up and the LED transmitting a reference signal is attached at the end of the blade. The LED is powered by a signal generator built affixed to the blade, as shown in Figure 9. Then, a translational velocity of the LED installed at the blade’s end assumed to be a moving vehicle can be formulated in terms of rpm and the length of the blade.

$$\begin{array}{cc} \; & 60\;\mathrm{rpm}=1\;\mathrm{rev}/s=2\pi \;\mathrm{rad}/s\;\\ \Rightarrow \; & 1\;\mathrm{rpm}=\frac{2\pi }{60}\;\mathrm{rad}/s\approx 0.10472\;\mathrm{rad}/s \end{array}$$

For the length of blade (R) of 1.0 m and the angular velocity of *x*, rpm measured by a tachometer, we find that the relative velocity is given by

$$\begin{array}{cc} V(\mathrm{linear}\;\mathrm{velocity}\;\mathrm{at}\;R) & =R\times \mathrm{angular}\;\mathrm{velocity}\\ & =R\times (x\;\mathrm{rpm}\times 0.10472)\\ \; & =(1.0\;m)\times (x\times 0.10472)\\ \; & \approx 0.10472\;x\;(m/s) \end{array}$$

The experiment is performed at nine different speeds to demonstrate the functionality of the sensor. Rates of 300, 350, 400, 450, 500, 550, 600, 650, 700 that correspond to tangential velocities (m/s) of 31.4, 36.7, 41.9, 47.1, 52.6, 57.6, 62.8, 68.1, 73.3, respectively, are used to demonstrate the prototype. Measurements at every 5-MHz increase in the input modulation frequency are used for the rate sensing.

**Figure 10:**
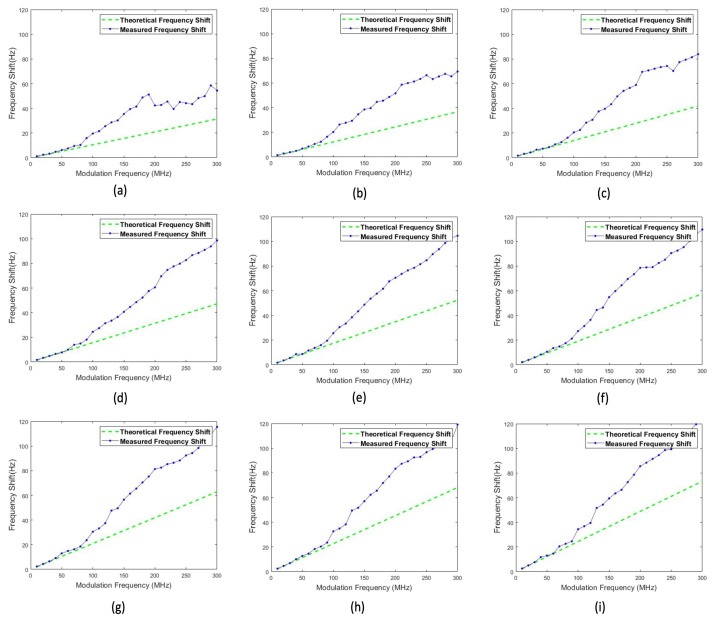
Result of the experiment reported at (**a**) 300 RPM (=linear velocity is 31.416 m/s). (**b**) 350 RPM (=linear velocity is 36.652 m/s). (**c**) 400 RPM (=linear velocity is 41.888 m/s). (**d**) 450 RPM (=linear velocity is 47.124 m/s). (**e**) 500 RPM (=linear velocity is 52.36 m/s). (**f**) 550 RPM (=linear velocity is 57.596 m/s). (**g**) 600 RPM (=linear velocity is 62.832 m/s). (**h**) 650 RPM (=linear velocity is 68.068 m/s). (**i**) 700 RPM (=linear velocity is 73.304 m/s). The green lines denote theoretical shifts and the blue lines denote the measured shifts.

The TIA sensor placed at the bench processes the optical signal coming from beacons to the RF port of the frequency mixer. The schematic in Figure 9 illustrates this setup. For reference, the other signal generator is connected to the LO port of the mixer and provides the frequency of the original transmitting signal. A digital oscilloscope is employed to read and display the output of the mixer. The oscilloscope features the ADC implementation with a 12-bit resolution for a signal visualization. Furthermore, the oscillator captures the measurement results over the sweep rate, and pulls the sampled signal data directly into a digital computer. Fast Fourier transform (FFT) is performed by the digital computer as post-processing so as to extract the frequency of the envelope signal, which is the frequency shift caused by relative motion. The obtained frequency shifts are plotted in Figure 10. The relative velocity can be derived from the measured frequency shift.

From Equation (2), the frequency shift is related to the velocity as,

(8) $$\Delta f\;\left(\mathrm{frequency}\;\mathrm{shift}\right)=\frac{V_{rel}\;f_{o}}{c}$$

where $V_{rel}$ is the relative velocity, and $f_{o}$ is the modulation frequency.

**Figure 11:**
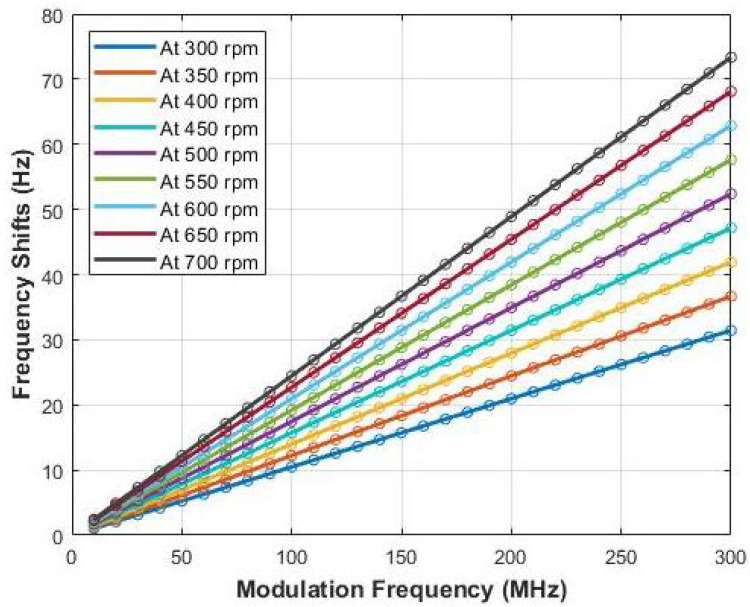
Theoretical (expected) frequency shifts at different RPMs.

Theoretically, frequency shifts should be directly proportional to both the rpm and the modulation frequency. Figure 11 shows the theoretical frequency shifts according to the rpm and modulation frequency. For each measurement, six trials are performed and their average value is reported as the confirmed measurement value.

The measured empirical results are now discussed.

### 4.2. Experimental Result

To demonstrate the capability of the sensor, the prototype circuit board and an LED beacon powered by a signal generator are tested at different modulating frequencies by operating the rotary experiment at different RPMs.

The results of the experiment reported in Figure 10 show similar trends. The errors in the frequency shift measurement become undesirable when the input modulation frequency exceeds 60 MHz. The modulation bandwidth of an LED is determined by the carrier lifetime and the parasitic capacitance of the LED [24]. It is also observed that the intensity of LED light decreases as the modulation frequency increases. After 100 MHz, energy emitted by the LED is quite low, contributing to a dramatic decrease in SNR of the signal detected by the TIA sensor. According to the manual description of the LED employed, this LED has a cut-off frequency of 70 MHz, which means the signal power is attenuated by 3 dB at 70 MHz and its optical signal power also drastically drops. This fact is verified by the empirical tests. Below 70 MHz, an excellent agreement between the frequency shift and the relative speed of the LED is observed. This verifies the conceptual principle of operation of the Doppler sensor over a variety of speeds. The test result in Figure 10 demonstrates the validity of the modulation bandwidth of the LED employed.

**Figure 12:**
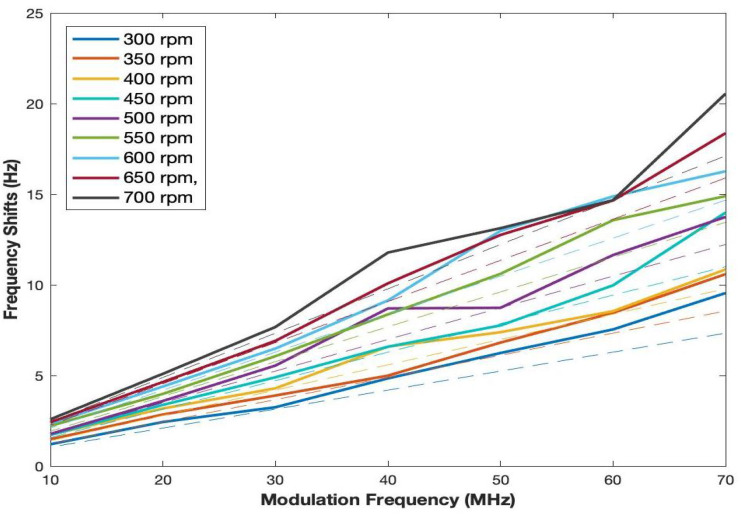
Measurements of frequency shifts for all RPMs up to the modulation frequency of 70 MHz (solid lines are measured values and dashed lines are real velocity generated by the experiment setup).

However, the frequency band of interest is narrowed down to 70 MHz. A comparison of measured frequency shifts with corresponding theoretical frequency shifts for modulation within 70 MHz is plotted in Figure 12.

### 4.3. Velocity Conversion

In this paper, the relative velocities between the target and the sensor are obtained by simple conversions from the frequency shift data, as shown in Equation (9).

(9) $$V_{rel}=c\;\frac{\Delta f}{f_{o}}$$

**Figure 13:**
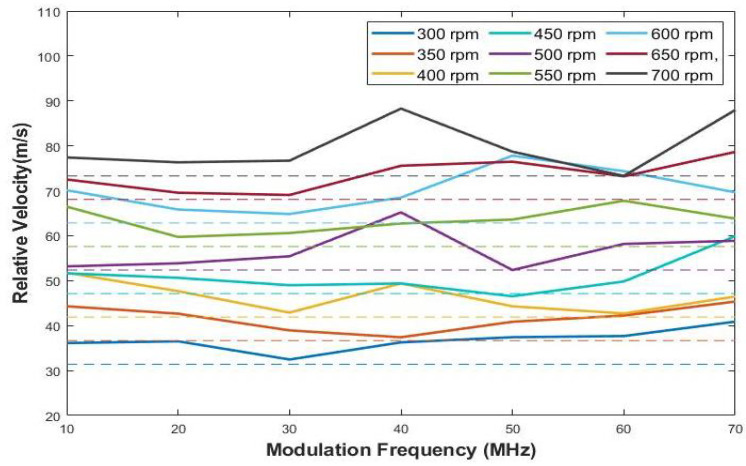
Comparison of estimated relative velocity to true relative velocity (solid lines are estimated values and dashed lines are the real velocity generated by the experimental setup).

The digital oscilloscope records the data of Figure 12 while sweeping across the oscilloscope screen. The data are saved in a CSV file so as to be employed in a digital computer by manual transfer. Recalling Equation (2) again, the boresight angle is set to be zero in this experiment by directly facing the sensor and light source. It is assumed that the structured beacons are affixed to the vehicle and the vehicle moves forward to the sensor with the tangential velocity of the experiment setup. According to Equation (9), the result of the conversion is illustrated in Figure 13.

The bias is offset by the average relative velocity obtained, assuming an initial zero bias. In practice, the precision information of the sensor is required to characterize the sensor performance. At each RPM, the residuals of the sensor output are observed, as reported in Figure 13. The deviations are caused by an intrinsic error added to the a small measurement error. We use the difference between the mean of measurements and true value in order to characterize an intrinsic error. By doing so, the effect of bias can be eliminated for each RPM. The average of error value for each RPM is presented in Table 1. The result in Figure 13 shows that the output of the sensor is consistent and usable if we consider the value of bias obtained. It is important to note that the unmodeled dynamics associated with the flapping of the blade and other misalignments contribute to the biases involved in this experiment. The intrinsic variability in the root mean square (RHS) errors of the biases incurred due to the experimental setup is difficult to quantify absolutely. The bias analysis attempts to provide an empirical agreement of this variability. The fact that the bias remains relatively stationary over an almost doubled linear velocity lends credence to the utility of the LED prototype for relative motions. Combining the specifications of LED and APD, and using Equation (4), the gains of the TIA and the noise are determined. For the proposed Doppler sensor, it is reported that $f_{z}$ is 200 kHz and $f_{p}$ is 1.53 MHz. According to the definition of noise gain in terms of a feedback factor, the presence of the feedback capacitance $C_{f}$ flattens the noise gain after $f_{p}$ [21]. If a capacitor is not installed in the feedback system, the noise gain keeps rising after $f_{z}$ and causes instability. Choosing the appropriate values of components is quite important for optimizing the performance of the circuit. However, it is not simple. Although the gain can be increased by higher feedback resistance, it lowers the bandwidth with smaller $f_{p}$. The TIA circuit is stable if a phase margin is greater than $45^{\circ }$. When a falling open-loop curve ($A_{ol}$) intersects at a rising 1/β curve, a 40-dB rate of closure occurs, developing a phase margin of less than $45^{\circ }$. To avoid an oscillation problem, $C_{f}$ should be increased to lower the $f_{p}$ so that it is developed on the left side of $A_{ol}$. However, it should be noted that noise gain is minimized if the region between $f_{z}$$f_{p}$ if $C_{f}$ is too large, and this region is reduced by increasing the $C_{f}$ [21,31]. The values of feedback components are carefully chosen to minimize the effect of noise and allow the beat signal to not be overshadowed by noise.

In comparison to a laser Doppler vibrometer that detects the displacement and velocity of a target using a laser beam as a source, the proposed sensor system employs low-cost and simpler design, which is designed for the part of a moving platform. For example, OptoMet’s HeNe single laser vibrometer [32] that shows very high accuracy up to 1.3 nm s${}^{-1}$ per Hz of vibration is hard to be loaded on a small vehicle because of numerable optical elements and a weight of 8 kg. The amplitude-modulated laser radar application developed by Mao et al. [33] also measures the target speed with high precision. Although our proposed sensor system has limited accuracy compared to the laser Doppler radar, it shows the greater range of detectable velocity and bandwidth owing to the simpler modulation of a LED.

## 5. Discussion

This paper presents a new method for estimating the velocity of a moving vehicle by combining the Doppler shift concept with optical signal modulation. A prototype opto-electronic sensor that implements proximity range rate measurements is devised. An LED light source, a photodetector, TIA circuit and a digital signal processor are used to build the prototype system. Experimental results demonstrate that the frequency shifts of the modulated light can be generated in proportion to a relative velocity between the sensor and light source. The accuracy of the proposed sensing system is found to be limited when the modulation is conducted beyond the bandwidth of the LED light source while the prototype bandwidth is significantly lower than that of a comparable system built using radio waves. The system shows promise reagarding the use of light in direct measurements of the Doppler range-rate of vehicles moving in relation to each other. A rotating experiment is developed to test the prototype Doppler range rate measurement system over a wide range of speeds. The frequency shift of the measured analog signals is examined by electronic instruments and a digital computer. Using the Doppler shift formula, the obtained frequency shift is converted to the relative velocity with acceptable accuracy. This result shows optimism regarding the use of a Doppler range-rate measurement system for relevant navigation applications.

## Figures and Tables

**Table 1:** Average of biases at RPMs (between 10 and 70 MHz).

RPM	Linear Velocity (m/s)	Bias (m/s)	Standard Deviation
300	31.416	5.3433	5.8233
350	36.652	5.0225	5.6661
400	41.888	4.5603	5.5447
450	47.124	3.8572	5.5050
500	52.36	4.3629	6.0143
550	57.596	5.9297	6.5108
600	62.832	7.3232	8.4667
650	68.068	5.5096	6.4170
700	73.304	6.5142	8.5092
